# The Low-Frequency Fluctuation of Trial-by-Trial Frontal Theta Activity and Its Correlation With Reaction-Time Variability in Sustained Attention

**DOI:** 10.3389/fpsyg.2020.01555

**Published:** 2020-07-14

**Authors:** Yao-Yao Wang, Li Sun, Yi-Wei Liu, Jia-Hui Pan, Yu-Ming Zheng, Yu-Feng Wang, Yu-Feng Zang, Hang Zhang

**Affiliations:** ^1^Institute of Psychological Sciences, College of Education, Hangzhou Normal University, Hangzhou, China; ^2^Center for Cognition and Brain Disorders, The Affiliated Hospital of Hangzhou Normal University, Hangzhou, China; ^3^Zhejiang Key Laboratory for Research in Assessment of Cognitive Impairments, Hangzhou, China; ^4^Institute of Mental Health, The Sixth Hospital, Peking University, Beijing, China

**Keywords:** sustained attention, EEG, frontal theta activity, reaction-time variability, trial-by-trial fluctuation, frequency-dependent fluctuation

## Abstract

Reaction-time variability is a critical index of sustained attention. However, researchers still lack effective measures to establish the association between neurophysiological activity and this behavioral variability. Here, the present study recorded reaction time (RT) and cortical electroencephalogram (EEG) in healthy subjects when they continuously performed an alternative responding task. The frontal theta activity and reaction-time variability were examined trial by trial using the measures of standard deviation (SD) in the time domain and amplitude of low-frequency fluctuation (ALFF) in the frequency domain. Our results showed that the SD of reaction-time variability did not have any correlation with the SD of trial-by-trial frontal theta activity, and the ALFF of reaction-time variability has a significant correlation with the ALFF of trial-by-trial frontal theta activity in 0.01–0.027 Hz. These results suggested the methodological significance of ALFF in establishing the association between neurophysiological activity and reaction-time variability. Furthermore, these findings also support the low-frequency fluctuation as a potential feature of sustained attention.

## Introduction

The capacity of sustained attention is of great importance. It refers to focusing on a certain task for a long period of time (Posner et al., 2014). Many occupations in our daily lives require a high level of sustained attention, e.g., driving vehicles, industrial control, and air traffic control (Aricò et al., 2016; Reinerman-Jones et al., 2016; Sebastiani et al., 2020). Declined sustained attention was documented in studies of several physiological states, e.g., alcoholism (Coles et al., 2002), sleep deprivation (Gunzelmann et al., 2009), and fatigue (Gunzelmann et al., 2011). Moreover, the deficit of sustained attention is usually identified as symptoms of various neuropsychiatric disorders, e.g., attention-deficit hyperactivity disorder (ADHD) (Barkley, 1997), and autism spectrum disorders (ASD) (Christakou et al., 2013). The practical importance of sustained attention, therefore, attracted interests from the research community, and numerous studies have been devoted to the behavioral and neurophysiological explorations of sustained attention.

Behavioral studies on sustained attention always employ examine stimulus-response tasks, e.g., alternative responding task (Helps et al., 2010), Go/NoGo task (Kirmizi-Alsan et al., 2006), and Eriksen flanker task (Castellanos et al., 2005). Subjects performing these tasks were requested to continuously detect stimulus, and their behavioral data of response and reaction time (RT) were recorded simultaneously. Several measures of the behavioral data were used to assess the level of sustained attention, e.g., error rate and intraindividual variability of RT. The error rate did not exhibit good test–retest reliability, and it was suggested to be more related with the response strategy (Liu et al., 2017; Steinborn et al., 2018). Therefore, the measure of intraindividual variability of RT was employed more extensively. For the individual, RT variability could be assessed by calculating the standard deviation of the reaction time (RT-SD) (Castellanos et al., 2005; Flehmig et al., 2007), and this measure in some studies was also standardized with the mean value of reaction time (RT-Mean) (Epstein et al., 2011; Tamm et al., 2012). RT-SD has good test–retest reliability (Liu et al., 2017), and their functional significance has been demonstrated by many clinical investigations. Observation from these investigations confirmed the linking between increased RT variability and the symptoms of sustained attention deficit, e.g., ADHD (Di Martino et al., 2008; Epstein et al., 2011), bipolar disorder (Brotman et al., 2009), and traumatic brain injury (TBI) (Burton et al., 2002). This functional significance of RT variability not only attracted increasing number of clinical trials (Rosch et al., 2013; James et al., 2016; Salum et al., 2019) but also promoted the neurophysiological explorations.

Cortical electroencephalogram (EEG) explorations have contributed many insightful evidences for the neurophysiological underpinning of sustained attention. Studies examining EEG identified several rhythmic activities, e.g., theta (4–8 Hz), alpha (8–14 Hz), and beta (14–30 Hz) (Buzsaki, 2006; Clayton et al., 2015), and some experimental evidences suggested that these EEG rhythmic activities were in response to distinct cognitive processes of sustained attention. Alpha was mainly in response to the inhibition of task-irrelevant processes (Klimesch et al., 2007; Uusberg et al., 2013), and beta could affect the attentional engagement to stimulus (Oswal et al., 2012; Coelli et al., 2015; Li et al., 2017; Shapiro et al., 2017). Theta was suggested to play a role in attentional control (Cohen and Donner, 2013; Cavanagh and Frank, 2014) and action monitoring process (Cavanagh et al., 2012; Pandey et al., 2016). Furthermore, studies attempt to establish the association between EEG rhythmic activity and behavior, and theta has been proven to be an important target (Helfrich et al., 2018; Fiebelkorn and Kastner, 2019). Increased theta activity, specifically the theta activity of the frontal brain regions, was identified in the conditions requiring higher level of sustained attention (Jensen and Tesche, 2002; Mitchell et al., 2008; Sauseng et al., 2010). More importantly, several studies reported that frontal theta activity successfully predicted the behavior during attention-demanding tasks and was related to prolonged task performance (Clayton et al., 2015; Helfrich et al., 2018). It was observed that increased frontal theta activity was in response of greater error rates and prolonged RTs (Boksem et al., 2005; Cavanagh and Shackman, 2015; Cooper et al., 2019); nevertheless, these results were not consistent across studies (Loo et al., 2004; van Driel et al., 2012; Wascher et al., 2014). Similar to RT variability, continuous recording of EEG activity typically exhibited a pattern of waxing and waning across trials of tests (Ratcliff et al., 2009; VanRullen et al., 2011). Inspired by this, recent studies explored the trial-by-trial fluctuation of EEG activity (Truccolo et al., 2002; Fox et al., 2006; McLoughlin et al., 2014; Adamo et al., 2015). Studies used the measure of SD to assess the frontal theta activity across different trials and directly examined the correlation between the SD of trial-by-trial frontal theta activity and RT-SD; however, they did not identify any significant results (McLoughlin et al., 2014).

It was worthy to note that an increasing number of studies documented the periodical variability of RT in sustained attention (Castellanos et al., 2005; Di Martino et al., 2008; Helps et al., 2011). This observation was firstly reported by Castellanos et al. (2005). They employed frequency-dependent analyses to examine RT fluctuation of children with ADHD when they perform a continuous stimulus–response test. It was found that the RT variability of the children with ADHD had peak amplitude around 0.05 Hz (about 20 s a cycle), and this peak amplitude of RT could be eliminated by using the medications of methylphenidate. These results, for the first time, revealed the periodical variability of RT for sustained attention. Since this periodical fluctuation was observed in the relatively low-frequency band (<0.2 Hz, >5 s/cycle), some studies employed the term amplitude of low-frequency fluctuation to depict this measure of reaction time (RT-ALFF). RT-ALFF as a measure of the frequency domain exhibited good test–retest reliability (ICC = 0.69) (Liu et al., 2017), and it has been repeatedly employed as a validity measure in examining the subjects with sustained attention deficits (Johnson et al., 2007; Vaurio et al., 2009; Karalunas et al., 2013). Studies usually investigated RT-ALFF in several frequency bands, including <0.01, 0.01–0.027, 0.027–0.073, and 0.073–0.167 Hz (Di Martino et al., 2008; Adamo et al., 2014, 2015). These frequency bands are defined by Penttonen and Buzsáki (2003) based on specific properties and physiological function. It has been found that RT-ALFF in three specific frequency bands (0.01–0.027 Hz; 0.027–0.073 Hz; 0.073–0.20 Hz) was strongly related to the ratings of ADHD symptoms (Mairena et al., 2012). These behavioral findings suggested that low-frequency fluctuation may be a feature of sustained attention, which encouraged us to investigate the ALFF of trial-by-trial frontal theta activity. We expected that the ALFF of trial-by-trial frontal theta activity could exhibit association with the ALFF of RT variability.

To verify this view, we performed an exploratory investigation. Data of RT and EEG activity were recorded simultaneously when subjects performing a sustained attention test. Trial-by-trial fluctuation of frontal theta activity and RT variability were examined with the measures of SD and ALFF (in the frequency bands of 0.01–0.027, 0.027–0.073, and 0.073–0.167 Hz). Then, EEG-behavior correlations were further assessed in each frequency band, respectively.

## Materials and Methods

### Participants

Seventy healthy participants (37 females; 21 ± 2 years old) were recruited in this study. All of the participants were right-handed, and no individual reported any history of brain injury or mental disorders. The RT data and EEG recording were collected from all participants during a continuously performed test, i.e., alternative responding task. According to the inclusion criteria of previous studies (Geurts et al., 2008; Liu et al., 2017), 4 participants (3 females) showing extreme mean RT values (longer than 3 SDs beyond the mean RT for all the participants) were excluded from subsequent analyses. At last, data from 66 participants (34 females; 21 ± 2 years old) were included in this study. All participants gave written informed consent prior to their participation. The study was approved by the Center for Cognition and Brain Disorders (CCBD) Ethics Committee of Hangzhou Normal University.

### Experimental Paradigm

The whole experiment involves two sessions of test, i.e., resting session and sustained attention task session, and the order of the two sessions was counterbalanced across all participants. Since the present study focused on trial-by-trial fluctuation, data from the resting session were not involved in the analysis. In the sustained attention task session, all participants performed the alternative responding task for 8 min (Helps et al., 2010). In the task, two kinds of stimuli, i.e., “>” and “<,” were pseudo-randomly presented for 500 ms interleaved with a fixation cross, and the inter-trial-interval (ITI) was 3000 ms. Each participant was instructed to determine the direction (left or right) of the arrow and to press “F” or “J” on the computer keyboard with the index finger. Before this session, a practice involving six trials was employed to ensure each participant was familiar with the procedure of the task.

### Electrophysiological Recording and Preprocessing

The EEG data were recorded using a 32-channel (Brain Products, Germany) extended 10–20 system montage. The original recording reference was positioned at FCz. A sampling rate of 500 Hz was used. The filter bandwidth at recording was 0.016–250 Hz. All impedances were kept below 5 kΩ. The EEG data from the task session was preprocessed using Vision Analyzer software (Brain Products, Germany). Specifically, the channel signals were firstly re-referenced to average reference. After applying a notch filter (50 Hz) and band pass filtering (0.1–70 Hz), eye movement artifacts were corrected using independent component analysis (ICA, Jung et al., 2000). Moreover, the stimulus-locked segments ranged from 0 to 3000 ms according to the ITI of the task. If the amplitude of the EEG data exceeded ± 100 mV at any electrode, a segment of 3 s around this artifact was excluded from further analyses; lastly, an average number of 158 (range from 137 to 160) artifact-free trials out of a total of 160 trials were available.

### Data Analysis

#### Behavioral Data Analyses

Behavioral measures, including several conventional measures and RT-ALFF, were calculated. The conventional measures including RT-Mean, RT-SD, and error rate (including commission error and omission error) were firstly acquired from each participant. Then, RT-ALFF was assessed through the following steps: (1) Missing and anticipatory responses (RT < 100 ms) were interpolated by linear interpolation to reconstruct an integrated time series. (2) The RT time series (divided by RT-Mean) were transformed from the time domain to the frequency domain through fast Fourier transformation (FFT), and the amplitude at each frequency point was obtained. (3) RT-ALFF was calculated as the mean amplitude in a fixed frequency band. The examinable frequency band was 0.002–0.167 Hz according to Nyquist’s sampling theorem (sampling rate is 0.33 Hz corresponding to the ITI of the task). RT-ALFF was calculated in three sub-frequency bands including 0.01–0.027, 0.027–0.073, and 0.073–0.167 Hz, which were widely explored in previous studies (Di Martino et al., 2008; Adamo et al., 2015).

#### Electrophysiological Data Analyses

**FIGURE 1:**
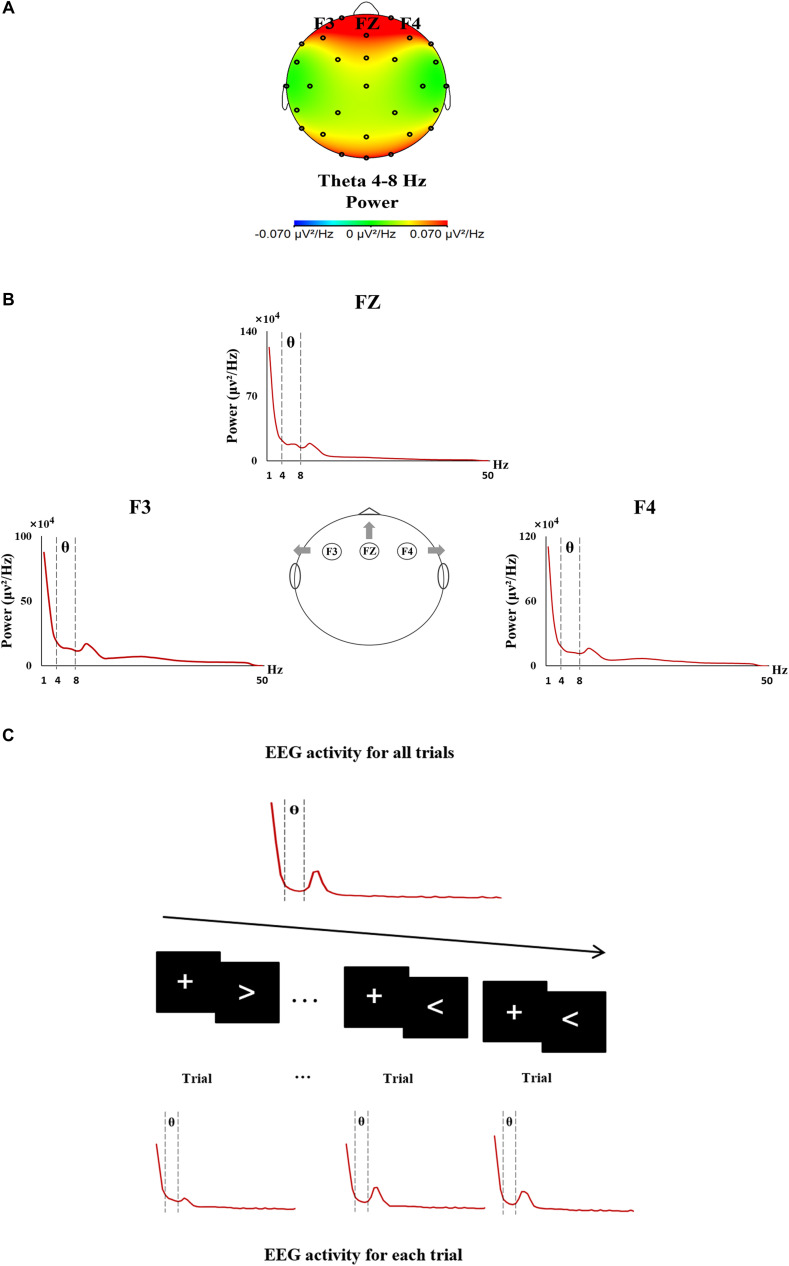
The procedure of EEG data analyses. **(A)** EEG topographic map showing the theta activity mainly located in the frontal area during the sustained attention task. **(B)** Frontal theta EEG activity was analyzed from 4 to 8 Hz at F3, FZ, and F4. **(C)** The procedure of the trial-by-trial analysis of EEG activity.

Electroencephalogram recording of the whole testing was analyzed through FFT, and the theta activity (4–8 Hz) mainly located in the frontal area was acquired at the electrodes of F3, FZ, and F4, respectively (Figures 1A,B). In the same way, trial-by-trial theta activity of each electrode was acquired based on the EEG recording of each single trial (each trial lasting 3000 ms) (Figure 1C).

**FIGURE 2:**
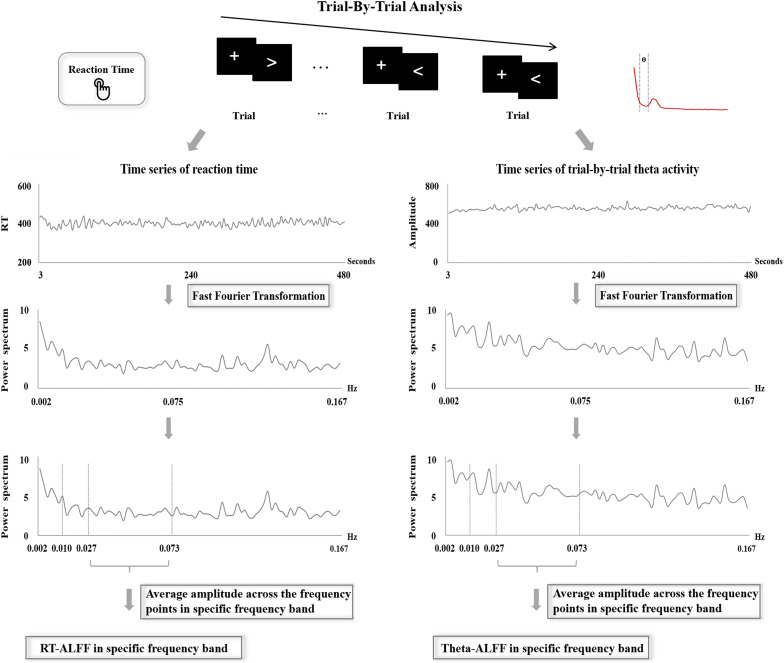
The procedure of the frequency-dependent analysis of reaction time and trial-by-trial theta activity.

Trial-by-trial theta activity (F3, FZ, and F4) was examined through two measures, Theta-SD and Theta-ALFF. For each frontal electrode, Theta-SD was calculated as the SD value of theta activity across all available trials. Theta-ALFF for each frontal electrode was assessed using the same analysis procedure of RT-ALFF (see details in Figure 2). Concretely, the theta activity of missing trials was replaced using linear interpolation between the theta activity of adjacent trials. Then, the time series of theta activity were divided by the mean value and were further transformed from the time domain to the frequency domain through FFT. At last, the average amplitude for a fixed frequency band was calculated as Theta-ALFF. Here, Theta-ALFF was examined in three frequency bands, i.e., 0.01–0.027, 0.027–0.073, and 0.073–0.167 Hz, corresponding to the analysis procedure of RT-ALFF. All of the analyses were implemented through our own MATLAB code.

#### Behavior-EEG Correlation Analyses

The correlations between behavioral measures and EEG measures were examined using Kendall rank-correlation analysis, since the data is not normally distributed (Shapiro–Wilk test, *p*-values < 0.045). The extreme values (longer than 3 SDs beyond the mean value of measure for all the participants) of RT data and EEG data were not involved in the analyses. The correlations between RT-SD and Theta-SD were first investigated, and then the correlations between RT-ALFF and Theta-ALFF were assessed in the frequency bands (0.01–0.027, 0.027–0.073, and 0.073–0.167 Hz). Moreover, we also examined the correlation between theta activity of whole testing and the above RT measures. All of these correlation analyses were performed using IBM SPSS Statistics (Version 20.0).

## Results

### Behavioral Results

Descriptive statistics of conventional behavioral measures were depicted in Table 1, including RT-Mean, RT-SD, commission error rate, and omission error rate, and the measures of RT-ALFF in all frequency bands are shown in Table 2.

**TABLE 1:** Conventional behavioral measures for the task performance.

**Behavioral measures**	**Mean ± SD (*N* = 66)**
RT-Mean (ms)	401.07 ± 39.88
RT-SD (ms)	63.87 ± 17.09
Commission error rate (%)	1.78 ± 1.75
Omission error rate (%)	0.42 ± 0.81

**TABLE 2:** RT-ALFF in each frequency band for the task performance.

**Frequency bands**	**RT-ALFF**
	**Mean ± SD (*N* = 66)**
0.01–0.027 Hz	1.86 ± 0.61
0.027–0.073 Hz	1.69 ± 0.38
0.073–0.167 Hz	1.71 ± 0.37

### Correlations Between RT-SD and Theta-SD

**FIGURE 3:**
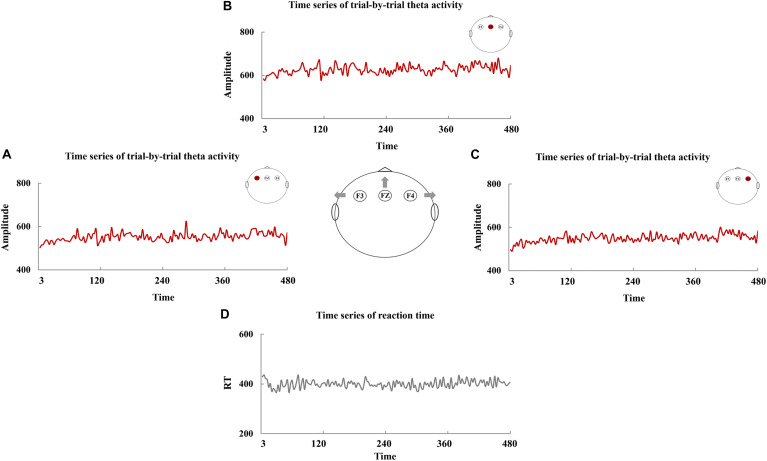
The time series of trial-by-trial theta activity and reaction time (RT). The time series of trial-by-trial theta activity at F3 **(A)**, FZ **(B)**, and F4 **(C)** and the time series of RT **(D)**.

The time series of RT are manifested in Figure 3D, and Figures 3A–C exhibited the time series of trial-by-trial theta activity at F3, FZ, and F4. The values of Theta-SD are shown in Table 3. The correlations between RT-SD and Theta-SD were assessed, and no significant correlation between RT-SD and Theta-SD was identified (each *r* < 0.01, *p* > 0.87) (Table 3). Moreover, no significant correlation between the theta activity of whole testing and RT-SD was identified (each *r* < 0.05, *p* > 0.56).

**TABLE 3:** Theta-SD and its correlations (Kendall rank correlation) with RT-SD.

**Electrodes**	**Theta-SD**	**Correlations with RT-SD**	
	**Mean ± SD (*N* = 66)**	***r***	***p***
F3	123.58 ± 56.95	0.01	0.87
FZ	140.04 ± 61.93	0.01	0.90
F4	120.05 ± 50.90	–0.003	0.97

### Correlations Between RT-ALFF and Theta-ALFF

**FIGURE 4:**
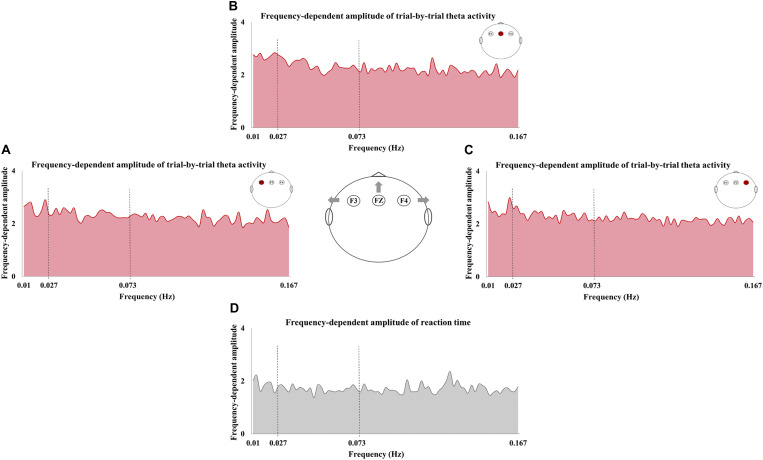
The frequency-dependent amplitude of trial-by-trial theta activity and reaction time (RT). The amplitude of trial-by-trial theta activity at F3 **(A)**, FZ **(B)**, and F4 **(C)** and the amplitude of RT fluctuation **(D)** in the sampled frequencies (0.01–0.167 Hz).

The frequency-dependent amplitude of reaction-time fluctuation is shown in Figure 4D, and Figures 4A–C exhibited the frequency-dependent amplitude of trial-by-trial theta activity at F3, FZ, and F4. Moreover, the values of Theta-ALFF in each frequency band are shown in Table 4.

**TABLE 4:** Theta-ALFF in each frequency band.

**Frequency bands**	**Theta-ALFF**		
	**Mean ± SD**	**Mean ± SD**	**Mean ± SD**
	**(F3, *N* = 66)**	**(FZ, *N* = 66)**	**(F4, *N* = 66)**
0.01–0.027 Hz	2.57 ± 0.98	2.73 ± 1.09	2.54 ± 0.93
0.027–0.073 Hz	2.35 ± 0.64	2.35 ± 0.64	2.32 ± 0.66
0.073–0.167 Hz	2.17 ± 0.60	2.17 ± 0.47	2.15 ± 0.58

**FIGURE 5:**
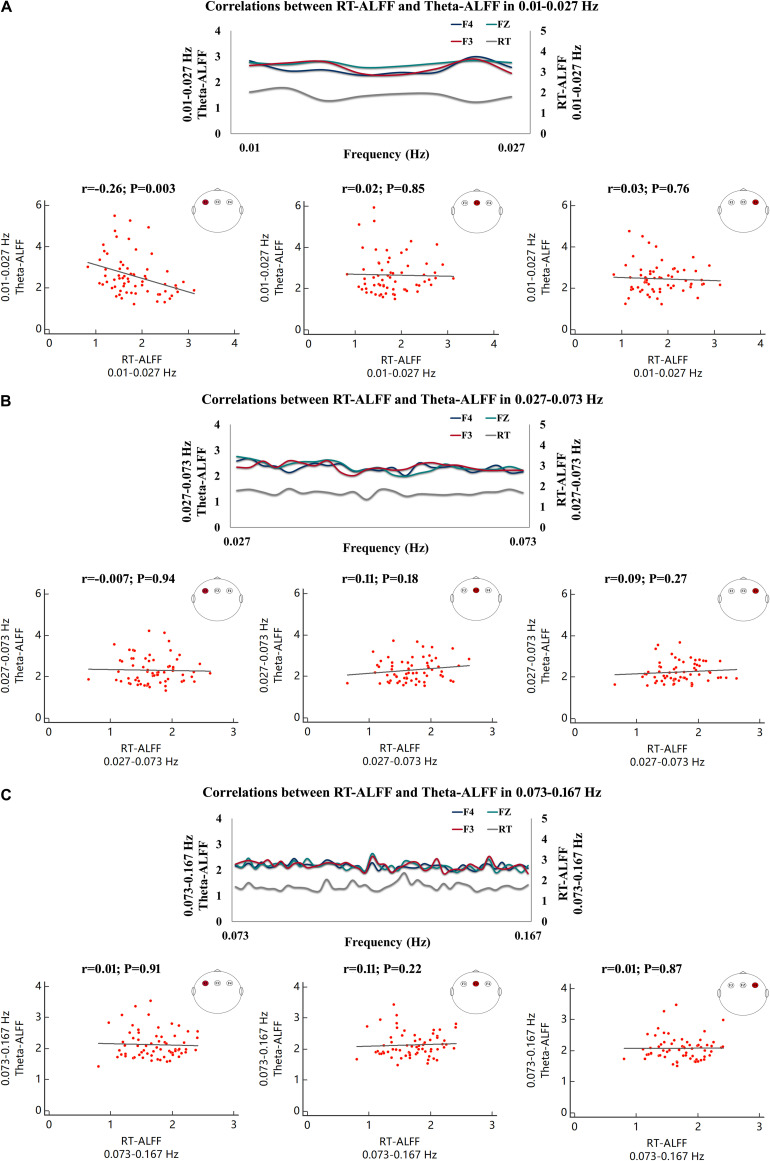
Correlations between RT-ALFF and Theta-ALFF in each frequency band. The correlation (Kendall rank correlation) between RT-ALFF and Theta-ALFF (at the electrodes of F3, FZ, and F4) is shown in 0.01–0.027 Hz **(A)**, 0.027–0.073 Hz **(B)**, and 0.073–0.167 Hz **(C)**. The amplitude of reaction time fluctuation and trial-by-trial theta activity (at the electrodes of F3, FZ, and F4) were also depicted in the frequencies of 0.01–0.027 Hz **(A)**, 0.027–0.073 Hz **(B)**, and 0.073–0.167 Hz **(C)**.

As shown in Figure 5, a significant correlation was observed in 0.01–0.027 Hz, and RT-ALFF was negatively correlated with Theta-ALFF at F3 (*r* = −0.26, *p* = 0.003, *p* < 0.05 after Bonferroni correction for three bands across three electrodes), not at F4 and FZ (Figure 5A). Then, the frequency band of 0.01–0.027 Hz was divided into two sub-frequency bands, i.e., 0.01–0.019 and 0.020–0.027 Hz. Correlations between RT-ALFF and Theta-ALFF could be reserved in the two bands (0.01–0.019 Hz, *r* = −0.22, *p* = 0.009 and 0.020–0.027 Hz, *r* = −0.22, *p* = 0.01).

No other significant correlations were found between RT-ALFF and Theta-ALFF in the frequency bands of 0.027–0.073 and 0.073–0.167 Hz (each *r* < 0.11, *p* > 0.18) (Figures 5B,C). Moreover, no significant correlation between the theta activity of whole testing and RT-ALFF in any frequency bands was identified (each *r* < 0.06, *p* > 0.46). In addition, the trial-by-trial ALFF of delta (1–4 Hz), alpha (8–14 Hz), beta (14–30 Hz), and gamma (30–50 Hz) and its correlation with RT-ALFF in each frequency band was examined separately, and no significant correlation was observed (each *r* < 0.13, *p* > 0.12) (see details in Supplementary Table S2).

## Discussion

The present study explored the association between trial-by-trial fluctuation of frontal theta activity and RT variability. Measures of SD and ALFF were used to establish this association in the time domain and frequency domain, respectively. It was observed that RT variability did not show any relationship with the trial-by-trial frontal theta activity when we used SD as the measure. In contrast, a frequency-dependent correlation between RT variability and trial-by-trial frontal theta activity was revealed by the measure of ALFF. These results provided a methodological insight for future studies on the neural underpinning of sustained attention.

Reaction-time variability was considered as a critical index of the level of sustained attention (Betts et al., 2006; Flehmig et al., 2007). Albeit intensive investigation, measures for establishing the association between the RT variability and neurophysiological activity was lacking (Rabbi et al., 2009; Clayton et al., 2015; Fortenbaugh et al., 2017; O’Halloran et al., 2018). It is always failed to identify significant results when the researcher directly examined the correlation between EEG activity, e.g., beta, theta, and RT variability (Buyck and Wiersema, 2015). Similarly, the present study also did not find any significant association between theta activity at each electrode and RT variability. Experimental observations indicated that the EEG activity always fluctuates across trials in a similar way as RT variability (Ratcliff et al., 2009; VanRullen et al., 2011). Thus, recent EEG explorations highlighted the trial-by-trial fluctuation of EEG activity (Fox et al., 2006; McLoughlin et al., 2014; Adamo et al., 2015). These studies mostly focused on the frontal theta activity because experimental evidences indicated the functional role of frontal theta activity in attentional control (Cohen and Donner, 2013; Cavanagh and Frank, 2014; McLoughlin et al., 2014). Consistent with their finding, the present study did not identify any significant correlation results when using SD to assess the RT variability and trial-by-trial fluctuation of frontal theta activity (McLoughlin et al., 2014). Methodically, SD is a measure of time domain reflecting the overall fluctuation of the testing data. It is worthy to note, however, that RT variability for sustained attention may occur periodically (Castellanos et al., 2005; Di Martino et al., 2008; Helps et al., 2011). Overall fluctuation potentially masked the periodical variability. This is a possible explanation for the findings on SD, and it further encouraged us to examine the trial-by-trial frontal theta activity and RT variability in frequency domain.

Clinical investigations indicated that children with ADHD could show increased RT variability in the low-frequency band (<0.2 Hz) (Castellanos et al., 2005; Karalunas et al., 2013). This observation was confirmed by many studies (Johnson et al., 2007; Di Martino et al., 2008; Vaurio et al., 2009; Helps et al., 2011), and these evidences indicated that the low-frequency fluctuation was a potential feature of sustained attention. Thus, the association between trial-by-trial frontal theta activity and RT variability may be frequency-dependent. This idea was confirmed by our findings of the ALFF analyses. RT-ALFF exhibited a significant correlation with the Theta-ALFF in 0.01–0.027 Hz. Behaviorally, frequency-dependent RT variability has been linked with the attention lapse (Castellanos et al., 2005; Johnson et al., 2007; Adamo et al., 2014). It has been reported that RT-ALFF in 0.01–0.027 Hz could be used as a predictor to explain scale ratings of inattention of ADHD (Mairena et al., 2012). Here, our results may provide some preliminary evidences for understanding the neural underpinning of these behavioral observations. Nevertheless, the functional significance of the frequency-dependent fluctuation of frontal theta activity required to be further clarified. Notably, the frequency-dependent correlation in the present study had spatial specificity, and the correlation was only identified at the left frontal electrode (F3). The importance of frontal theta activity in sustained attention has been discussed (Missonnier et al., 2006; Gongora et al., 2015); however, the functional differences between the theta activity of the left and right frontal areas were less addressed in these EEG studies. It was reported that theta activity in the frontal brain areas may function in attention maintenance (Cavanagh and Frank, 2014; Wascher et al., 2014). Here, we found that the greater Theta-ALFF at the left electrode (F3) was negatively correlated with lower RT-ALFF. This finding reinforced the role of frontal theta activity in attention maintenance. Source location analysis further linked these findings with previous neuroimaging studies. The origin of the theta activity was implicated in the ventral medial frontal gyrus (vmPFC) (see details in the Supplementary Material), and this region serving as a role of state monitoring has been intensively reported by the functional magnetic resonance imaging (fMRI) studies on sustained attention (Ridderinkhof et al., 2004; Hasenkamp et al., 2012; Sepede et al., 2012; Clayton et al., 2015). Nevertheless, the EEG data, collected from 32 electrodes, could not provide robust localization results (Michel et al., 2004). Therefore, the linking between theta activity, vmPFC, and behavior variability should be examined with more experiments in the future. Moreover, significant correlation only appears on the left frontal electrode (F3). This spatial specificity suggested the functional differences between the theta activity of left and right areas, which should be taken into account by further explorations.

The present study sheds light on the low-frequency fluctuation of trial-by-trial EEG activity. Actually, the low-frequency fluctuation of brain activity has been documented in many previous studies. However, most of these explorations focused on fMRI but not on EEG activity (Sonuga-Barke and Castellanos, 2007; Zang et al., 2007). The findings of the present study supported the feature of low-frequency fluctuation of sustained attention, and thus, ALFF as a measure of frequency domain will subserve the establishment of the association between RT variability and EEG activity. Notably, the present study focused on EEG rhythmic activity, and we believe that it is also an interesting issue whether low-frequency fluctuation could be identified in other EEG components, e.g., event-related potential (ERP). Adamo et al. (2015) have attempted to explore this issue, and they reported that the trial-by-trial variability of P3 and RT variability coupled at 0.073–0.167 Hz when subjects performed the Go/NoGo tasks (Adamo et al., 2015). However, their findings may be confounded by many experimental factors. For example, the restriction of the signal-to-noise ratio makes it challengeable to extract robust ERP from data of a single trial. ERP is usually acquired from tasks with jitter in ITI, and the jitter results in more frequency complexity. So, this issue still needs to be examined with well-designed experiments in future studies. Moreover, the low-frequency fluctuation of trial-by-trial EEG activity, in the present study, is derived from the specific task, i.e., alternative responding task. This task has obvious advantages for frequency-dependent analysis. It has continuous response, and the time series does not need to be reconstructed statistically as is required by many non-responding trials in more complex tasks. Behavioral evidences suggested that the task complexity could induce the variation in the frequency band of fluctuation for sustained attention (Johnson et al., 2007; Di Martino et al., 2008; Helps et al., 2011; Karalunas et al., 2013; Salum et al., 2019). Therefore, further experimentation with different tasks is required to verify the band specificity of our findings.

Several limitations existed in the present study. First, the subjects were all healthy college students; further studies on the subjects with attention deficits were required to examine whether the trial-by-trial fluctuation of frontal theta activity has clinical significance. Second, the ITI of the task paradigm for the current study was 3000 ms, and thus, the frequencies that we could analyze only ranged from 0.002 to 0.167 Hz, according to the Nyquist sampling theorem. The explorations on higher frequencies (>0.167 Hz) are necessary, and tasks with fast behavior recording may be helpful for this issue. Third, our finding has spatial specificity in F3, and this electrode is spatially close to the original reference, FCz. This reference during data collection is a fixed setting for the EEG device. Therefore, it remains to be investigated whether this setting affects our findings in F3.

## Conclusion

The present study is an exploratory investigation and, for the first time, reported that the correlation between RT variability and trial-by-trial frontal-theta activity was frequency-dependent. ALFF as a measure of the frequency domain exhibited methodological significance in establishing the association between RT variability and EEG activity. These findings supported the low-frequency fluctuation as a feature of sustained attention. Further explorations on this feature may facilitate the understanding of the neuro underpinning of sustained attention.

## Data Availability Statement

The datasets generated for this study are available on request to the corresponding author.

## Ethics Statement

The studies involving human participants were reviewed and approved by the Center for Cognition and Brain Disorders (CCBD) Ethics Committee of the Hangzhou Normal University. The patients/participants provided their written informed consent to participate in this study. Written informed consent was obtained from the individual(s) for the publication of any potentially identifiable images or data included in this article.

## Author Contributions

HZ conceived and designed the experiment. Y-YW, Y-WL, and J-HP collected the data. Y-YW, Y-WL, J-HP, and Y-MZ performed the data analysis. LS, Y-FW, and Y-FZ provided advice on the analysis and interpretation of the results. Y-YW and HZ wrote the manuscript. All authors contributed to the article and approved the submitted version.

## Conflict of Interest

The authors declare that the research was conducted in the absence of any commercial or financial relationships that could be construed as a potential conflict of interest.
