# Supplementary material for: The Low-Frequency Fluctuation of Trial-by-Trial Frontal Theta Activity and Its Correlation With Reaction-Time Variability in Sustained Attention
Source: Front Psychol. 2020 Jul 14;11:1555. doi: 10.3389/fpsyg.2020.01555 (PMC7381245; doi:10.3389/fpsyg.2020.01555)
Supplement: Supplementary file 1 [file Data_Sheet_1.pdf]

Supplementary Materials

1 Methods

1.1 Analyses of Delta, Alpha, Beta and Gamma

The preprocessed EEG data of each single trial was first transformed from time domain to frequency domain. A Fast Fourier Transformation (FFT) was performed, and the EEG data from three frontal electrodes (F3, FZ and F4) were divided into four EEG activity: delta (1-4 Hz), alpha (8-14 Hz), beta (14-30 Hz) and gamma (30-50 Hz) (Clayton et al., 2015; Mantini et al., 2007).

Trial-by-trial activity of delta, alpha, beta, and gamma was examined through two measures, SD and ALFF. For each frontal electrode, trial-by-trial SD of each EEG activity was calculated as the SD value of each EEG activity across all available trials. The trial-by-trial ALFF of these EEG activities for each frontal electrode was assessed through the following steps: (1) Continuous time series of each EEG activity was generated by using linear interpolation between adjacent observations to replace the missing trials; (2) The time series of each EEG activity was divided by the mean value and was further transformed from time domain to frequency domain through FFT separately; (3) At last, the average amplitude for a fixed frequency band was calculated as trial-by-trial ALFF of each EEG activity separately. Here, trial-by-trial ALFF of each EEG activity was examined in three frequency bands, i.e., 0.01–0.027 Hz, 0.027–0.073 Hz and 0.073–0.167 Hz corresponding to the analysis procedure of RT-ALFF (Adamo et al., 2015; Di Martino et al., 2008). All of the analyses were implemented through our own Matlab code.

1.2 Behavior-EEG correlation analyses

The correlations between behavioral measures and EEG measures were examined using Kendall rank correlation, because the data were not normally distributed (Shapiro-Wilk test, p-values<0.047). The extreme values (longer than 3 standard deviations beyond the mean value of measure for all the participants) of RT data and EEG data were not involved in the analyses. The correlations between RT-SD and trial-by-trial SD of each EEG activity were first investigated separately, and then, the correlations between RT-ALFF and trial-by-trial ALFF of each EEG activity were assessed in the frequency bands (0.01–0.027 Hz, 0.027–0.073 Hz and 0.073–0.167 Hz) separately. All of these correlation analyses were performed using IBM SPSS Statistics (Version 20.0).

1.3 Source localization analysis of theta activity

On the basis of the scalp-recorded electric potential distribution (32-channel EEG acquisition system), sLORETA (standardized low-resolution brain electromagnetic tomography) was used to compute the cortical three-dimensional distribution of current density of theta activity. The sLORETA method finds a particular solution to the non-unique EEG inverse problem by assuming similar activation of neighboring neuronal sources, followed by an appropriate standardization of the current density, producing images of electric neuronal activity without localization bias (Pascual-Marqui, 2002). Computations were made in a realistic head model (Fuchs et al., 2002), using the MNI152 template (Mazziotta et al., 2001), with the three-dimensional solution space restricted to cortical grey matter. The intracerebral

volume is partitioned in 6239 voxels at 5 mm spatial resolution. Anatomical labels are reported using an appropriate correction from MNI to Talairach space (Brett et al., 2002). Hence, sLORETA images represent the electric activity at each voxel in neuroanatomic Talairach space (Talairach and Tournoux, 1988) as the squared standardized magnitude of the estimated current density.

The electrode coordinates were created from the electrode locations using the original recording montage. A transformation matrix was created using the electrode coordinates. The averaged waveforms (averaged from single trials, i.e., 3000ms) were converted and saved into ASCII values for each subject. And the sLORETA values for these averaged waveforms were computed for each subject separately using ASCII values, electrode coordinates, and the transformation matrix.

The grand average theta band sLORETA images were computed by calculating in a first step the sLORETA solution for each subject, and in a second step by averaging the current density values across all subjects for theta band (4-8 Hz).

2 Results

2.1 Correlations between RT-SD and the SD of EEG activities

The trial-by-trial SD of delta, alpha, beta and gamma and its correlation with RT-SD was examined, no significant correlation was observed (each r<0.08, p>0.37) (TableS1).

TableS1. Correlations between RT-SD and the SD of EEG activities

EEG activities	Electrodes Locations		
	**F3**	**FZ**	**F4**
	r (p)	***r (p)***	***r (p)***
**Delta**	0.05(0.54)	0.06(0.51)	0.03(0.75)
**Alpha**	0.02(0.83)	0.04(0.62)	0.07(0.42)
**Beta**	-0.007(0.94)	-0.05(0.60)	0.03(0.72)
**Gamma**	0.05(0.56)	-0.04(0.66)	0.08(0.37)

2.2 Correlations between RT-ALFF and the ALFF of EEG activities

The trial-by-trial ALFF of delta, alpha, beta and gamma and its correlation with RT-ALFF in three frequency bands (0.01–0.027 Hz, 0.027–0.073 Hz and 0.073–0.167 Hz) was examined separately, no significant correlation was observed in any frequency band (each $r<0.13$, $p>0.12$) (TableS2).

TableS2. Correlations between RT-ALFF and the ALFF of EEG activities in each frequency band

**EEG activities**	**Electrodes Locations**		
	**F3**	**FZ**	**F4**
	r (p)	***r (p)***	***r (p)***
	**Correlations with RT-ALFF in 0.01-0.027 Hz**		
**Delta**	0.03(0.73)	0.05(0.55)	0.04(0.66)
**Alpha**	-0.03(0.74)	-0.03(0.75)	-0.06(0.45)
**Beta**	-0.13(0.12)	-0.05(0.57)	-0.10(0.23)
**Gamma**	-0.07(0.40)	-0.03(0.69)	-0.11(0.19)
	**Correlations with RT-ALFF in 0.027-0.073 Hz**		
**Delta**	0.008(0.93)	0.04(0.64)	0.008(0.93)
**Alpha**	0.03(0.77)	0.11(0.20)	0.06(0.51)
**Beta**	-0.05(0.59)	-0.04(0.66)	-0.05(0.58)
**Gamma**	0.10(0.23)	0.09(0.32)	0.09(0.28)
	**Correlations with RT-ALFF in 0.073-0.167 Hz**		
**Delta**	0.04(0.67)	0.07(0.43)	0.08(0.37)
**Alpha**	0.05(0.57)	0.08(0.33)	0.03(0.75)
**Beta**	-0.04(0.61)	0.07(0.39)	-0.06(0.51)
**Gamma**	0.06(0.53)	0.03(0.73)	-0.01(0.91)

2.3 sLORETA results

In order to localize the source of theta activity, sLORETA was applied. sLORETA mainly localized the theta activity to medial frontal gyrus (as shown in FigureS1).

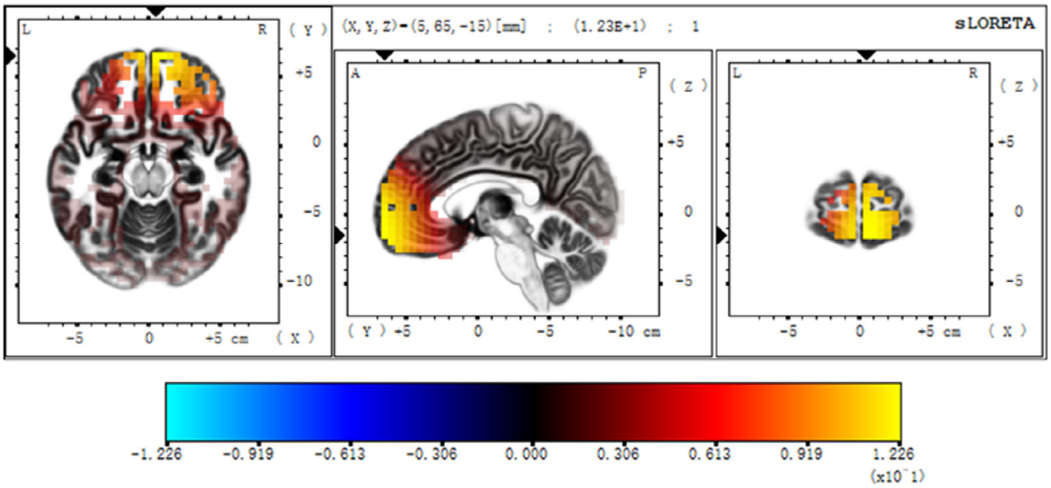

FigureS1 Localization of Theta band with sLORETA. Grand average Theta sLORETA images showing standardized current density maxima of all subjects (n=66). Each map consists of axial, sagittal, and coronal planes showing the same activation areas. Maxima are color coded as yellow. Yellow color indicates local maxima of the theta activity in Brodmann area 11 (X=5, Y=65, Z=-15). L=left; R=right; X/Y/Z=MNI coordinates (Montreal Neurological Institute); A=anterior; P=posterior.

References

Clayton, M. S., Yeung, N., & Kadosh, R. C. (2015). The roles of cortical oscillations in sustained attention. *Trends in cognitive sciences*, *19*(4), 188-195. doi: 10.1016/j.tics.2015.02.004

Mantini, D., Perrucci, M. G., Del Gratta, C., Romani, G. L., & Corbetta, M. (2007). Electrophysiological signatures of resting state networks in the human brain. *Proceedings of the National Academy of Sciences*, *104*(32), 13170-13175. doi: 10.1073/pnas.0700668104

Adamo, N., Baumeister, S., Hohmann, S., Wolf, I., Holz, N., Boecker, R., Laucht, M.,

Banaschewski, T., & Brandeis, D. (2015). Frequency-specific coupling between trial-to-trial fluctuations of neural responses and response-time variability. *Journal of neural transmission*, *122*(8), 1197-1202. doi: 10.1007/s00702-015-1382-8

Di Martino, A., Ghaffari, M., Curchack, J., Reiss, P., Hyde, C., Vannucci, M., Petkova, E., Klein, D.F., & Castellanos, F. X. (2008). Decomposing intra-subject variability in children with attention-deficit/hyperactivity disorder. *Biological psychiatry*, *64*(7), 607-614. doi: 10.1016/j.biopsych.2008.03.008

Pascual-Marqui, R. D. (2002). Standardized low-resolution brain electromagnetic tomography (sLORETA): technical details. *Methods Find Exp Clin Pharmacol*, *24*(Suppl D), 5-12. Available online at: http://www.ncbi.nlm.nih.gov/pubmed/12575463

Fuchs, M., Drenckhahn, R., Wischmann, H., & Wagner, M. (1998). An improved boundary element method for realistic volume-conductor modeling. *IEEE Transactions on Biomedical Engineering*, *45*(8), 980-997. doi: 10.1109/10.704867

Mazziotta, J., Toga, A., Evans, A., Fox, P., Lancaster, J., Zilles, K., Woods, R., Paus, T., Simpson, G., Pike, B., Holmes, C., Collins, L., Thompson, P., MacDonald, D., Iacoboni, M., Schormann, T., Amunts, K., PalomeroGallagher, N., Geyer, S., Parsons, L., Narr, K., Kabani, N., Le Goualher, G., Boomsma, D., Cannon, T., Kawashima, R., & Mazoyer, B. (2001). A probabilistic atlas and reference system for the human brain: International Consortium for Brain Mapping (ICBM). *Philosophical Transactions of the Royal Society of London. Series B: Biological Sciences*, *356*(1412), 1293-1322. doi: 10.1098/rstb.2001.0915

Brett, M., Johnsrude, I. S., & Owen, A. M. (2002). The problem of functional localization in the human brain. *Nature reviews neuroscience*, *3*(3), 243-249. doi: 10.1038/nrn756

Talairach, J. (1988). Co-planar stereotaxic atlas of the human brain-3-dimensional proportional system. *An approach to cerebral imaging*. doi: 10.1017/s0022215100111879
